# Amino acid and mineral digestibility, bone ash, and plasma inositol is increased by including microbial phytase in diets for growing pigs

**DOI:** 10.1186/s40104-023-00953-x

**Published:** 2023-12-10

**Authors:** Liz Vanessa Lagos, Mike Richard Bedford, Hans Henrik Stein

**Affiliations:** 1 Division of Nutritional Sciences, University of Illinois, Urbana, IL 61801 USA; 2 AB Vista, Marlborough, SN8 4AN UK

**Keywords:** Bone ash, Inositol, Nutrient digestibility, Phytase, Phytate degradation, Pigs

## Abstract

**Background:**

The effect of microbial phytase on amino acid and energy digestibility is not consistent in pigs, which may be related to the phytase dosage or the adaptation length to the diet. Therefore, an experiment was conducted to test the hypotheses that increasing dietary phytase after an 18-day adaptation period: 1) increases nutrient and energy digestibility; 2) increases plasma P, plasma inositol, and bone ash of young pigs; and 3) demonstrates that maximum phytate degradation requires more phytase than maximum P digestibility.

**Results:**

Data indicated that increasing inclusion of phytase [0, 250, 500, 1,000, 2,000, and 4,000 phytase units (FTU)/kg feed] in corn-soybean meal-based diets increased apparent ileal digestibility (AID) of Trp (quadratic; *P* < 0.05), and of Lys and Thr (linear;* P* < 0.05), and tended to increase AID of Met (linear;* P* < 0.10). Increasing dietary phytase also increased AID and apparent total tract digestibility (ATTD) of Ca and P (quadratic; *P* < 0.05) and increased ATTD of K and Na (linear;* P* < 0.05), but phytase did not influence the ATTD of Mg or gross energy. Concentrations of plasma P and bone ash increased (quadratic; *P* < 0.05), and plasma inositol also increased (linear;* P* < 0.05) with increasing inclusion of phytase. Reduced concentrations of inositol phosphate (IP)6 and IP5 (quadratic; *P* < 0.05), reduced IP4 and IP3 (linear;* P* < 0.05), but increased inositol concentrations (linear;* P* < 0.05) were observed in ileal digesta as dietary phytase increased. The ATTD of P was maximized if at least 1,200 FTU/kg were used, whereas more than 4,000 FTU/kg were needed to maximize inositol release.

**Conclusions:**

Increasing dietary levels of phytase after an 18-day adaptation period increased phytate and IP ester degradation and inositol release in the small intestine. Consequently, increasing dietary phytase resulted in improved digestibility of Ca, P, K, Na, and the first 4 limiting amino acids, and in increased concentrations of bone ash and plasma P and inositol. In a corn-soybean meal diet, maximum inositol release requires approximately 3,200 FTU/kg more phytase than that required for maximum P digestibility.

## Background

Inclusion of microbial phytase in diets for swine and poultry has been practiced since the early 1990’s [1]. The standard level of inclusion ranges from 250 to 500 phytase units (FTU)/kg of feed, which is expected to release phytate-bound P from plant-based feed ingredients to reduce the amount of feed phosphate needed in the diet without compromising growth performance or bone mineralization [2]. Calcium is also released by phytase as a result of interactions between Ca and phytate [3]. Supplementation of phytase at doses above 1,000 FTU/kg, also known as super-dosing, has resulted in improvements in growth performance of growing pigs [4]. This improvement in animal performance has been attributed to a reduction in the anti-nutritional effects of phytate, and a subsequent increase in nutrient digestibility and inositol release [5]. Degradation of phytate produces lower phytate esters that precipitate with available cations and interact with proteins to a lesser extent than phytate [6]. However, results of in vitro experiments indicate that inositol phosphate (IP)4 and IP3 also inhibit pepsin activity [7], and degradation of phytate and lower phytate esters, therefore, is important for proper mineral and protein digestion. Data from broiler chickens demonstrated the beneficial effect of super-dosing of phytase on energy, amino acid (AA), and mineral digestibility [8], but data from pigs have not consistently demonstrated similar effects [9, 10].

Although positive effects of phytase on mineral digestibility in pigs is often observed [11–13], data indicate that increasing levels of phytase up to 3,000 FTU/kg has limited effects on AA and energy digestibility if a 5- or 7-day adaptation period to the experimental diets is used [14, 15]. However, it is not known if providing greater phytase doses and allowing more time for pigs to adapt to their assigned diets will result in increased AA and energy digestibility. Additionally, data demonstrating the effect of increasing levels of phytase in a corn-soybean meal (SBM) diet on phytate and phytate ester degradation in pigs are scarce [16]. Therefore, the objective of this experiment was to test the hypothesis that increasing inclusion of dietary phytase from 0 to 4,000 FTU/kg in corn-SBM diets fed to growing pigs allowed an 18-day adaptation period to diets results in the following outcomes: 1) increased apparent digestibility of minerals, AA, and energy; 2) increased concentrations of plasma P, plasma inositol, and bone ash; and 3) confirmation that maximum phytate degradation requires more phytase than what is needed to maximize P digestibility.

## Methods

The Institutional Animal Care and Use Committee at the University of Illinois reviewed and approved the protocol for this experiment. Pigs used in the experiment were the offspring of Line 359 boars and Camborough females (Pig Improvement Company, Hendersonville, TN, USA). 

### Animals, housing, and diets

A total of 36 castrated male pigs were equipped with a T-cannula (barrel length: 6 cm; inner diameter: 1.6 cm) in the distal ileum [17]. After 3 to 5 days of recovery, pigs (initial body weight: 11.0 ± 0.6 kg) were allotted to 1 of 6 diets in a completely randomized design with 6 replicate pigs per diet. Pigs were housed individually in 0.9 m × 1.8 m pens that had fully slatted concrete floors and were equipped with a feeder and a cup waterer in an environmentally controlled room. Water and feed were available at all times. The Experimental Animal Allotment Program [18] was used to allot pigs to experimental diets.

Corn, SBM, lactose, limestone, and monocalcium phosphate were procured from local suppliers (Table 1). Based on these ingredients, six diets were formulated by including 0, 250, 500, 1,000, 2,000, or 4,000 FTU/kg feed (Quantum Blue; AB Vista Feed Ingredients, Marlborough, UK) in each diet (Table 2). The phytase concentrate contained 5,000 FTU/g and was mixed with corn to prepare a premix that was included at 0.50% into each diet. Provisions of total Ca and standardized total tract digestible P were reduced by 0.16% and 0.11%, respectively, compared with requirement estimates (11 to 25 kg; [19]) to account for the expected release of Ca and P by phytase. All diets contained 0.40% chromic oxide as an indigestible marker, and vitamins and minerals other than Ca and P were included to meet or exceed the requirement. All diets were fed as mash and no further processing after mixing was conducted. A representative sample of 2 kg of each diet and ingredient was collected at the time of diet mixing.

**Table 1:** Analyzed composition of ingredients

Item	Ground maize	Soybean meal	Lactose	Calcium carbonate	Monocalcium phosphate
Gross energy, kcal/kg	3,829	4,193	3,591	-	-
Dry matter, %	85.53	88.27	94.96	99.99	93.88
Ash, %	1.70	7.47	0.30	89.68	81.35
Crude protein, %	5.91	45.60	-	-	-
AEE^a^, %	3.86	1.63	-	-	-
Ca, %	0.04	0.28	0.02	38.93	17.26
P, %	0.25	0.59	0.01	0.03	20.46
Phytate^b^, %	0.63	1.54	-	-	-
Phytate-bound P, %	0.18	0.43	-	-	-
Non-phytate P^c^, %	0.07	0.16	-	-	-

^a^*AEE* Acid hydrolyzed ether extract

^b^Phytate was calculated by dividing the concentration of phytate-bound P by 0.282 [20]

^c^Non-phytate P was calculated as the difference between total P and phytate-bound P

**Table 2:** Ingredient composition and analyzed composition of experimental diets^a^

Item	Phytase units (FTU)/kg feed					
	**0**	**250**	**500**	**1,000**	**2,000**	**4,000**
Ingredient, %						
Ground maize	49.70	49.20	49.20	49.20	49.20	49.20
Soybean meal (48% crude protein)	35.00	35.00	35.00	35.00	35.00	35.00
Lactose	10.00	10.00	10.00	10.00	10.00	10.00
Soybean oil	2.30	2.30	2.30	2.30	2.30	2.30
Calcium carbonate	0.95	0.95	0.95	0.95	0.95	0.95
Monocalcium phosphate	0.30	0.30	0.30	0.30	0.30	0.30
Sodium bicarbonate	0.32	0.32	0.32	0.32	0.32	0.32
L-Lys HCl (78% Lys)	0.28	0.28	0.28	0.28	0.28	0.28
DL-Met	0.12	0.12	0.12	0.12	0.12	0.12
L-Thr	0.08	0.08	0.08	0.08	0.08	0.08
Sodium chloride	0.40	0.40	0.40	0.40	0.40	0.40
Chromic oxide	0.40	0.40	0.40	0.40	0.40	0.40
Vitamin mineral premix^b^	0.15	0.15	0.15	0.15	0.15	0.15
Phytase-corn premix^c^	-	0.50	0.50	0.50	0.50	0.50
Total	100	100	100	100	100	100
Analyzed values						
Gross energy, kcal/kg	3,987	3,956	4,003	3,976	3,991	3,995
Dry matter, %	87.95	87.86	87.95	87.81	87.78	87.75
Ash, %	5.35	5.04	5.06	5.22	5.27	5.28
Crude protein, %	19.42	19.59	19.40	19.07	19.72	20.16
AEE^d^, %	3.72	3.77	3.96	3.74	3.83	3.93
Amino acids, %						
Arg	1.38	1.30	1.32	1.29	1.28	1.34
His	0.55	0.52	0.53	0.52	0.51	0.53
Ile	0.95	0.91	0.91	0.89	0.87	0.91
Leu	1.71	1.64	1.68	1.65	1.61	1.68
Lys	1.45	1.34	1.36	1.36	1.33	1.36
Met	0.44	0.37	0.40	0.41	0.37	0.44
Phe	1.02	1.01	0.99	0.97	0.95	1.03
Thr	0.86	0.81	0.79	0.81	0.80	0.83
Trp	0.28	0.26	0.27	0.26	0.27	0.27
Val	1.04	0.98	0.99	0.97	0.96	0.99
Ca, %	0.58	0.56	0.55	0.55	0.57	0.60
P, %	0.43	0.44	0.42	0.42	0.42	0.43
Phytate bound-P, %	0.26	0.25	0.24	0.24	0.23	0.21
Phytase activity, FTU/kg	< 50	417	715	1,680	2,760	5,350
Phytate esters^e^, nmol/g dry matter						
IP6	15,225	12,920	13,732	15,242	14,540	12,005
IP5	1,964	1,831	1,851	2,029	1,790	1,700
IP4	244	228	275	273	282	444

^a^Diets were formulated to contain the following quantities of net energy, amino acids (expressed as standardized ileal digestible), Ca, and P: net energy, 2,473 kcal/kg; Lys, 1.23%; Met, 0.40%; Thr, 0.74%; Trp, 0.23%; Ca, 0.54%; P, 0.44%

^b^The vitamin–micromineral premix provided the following quantities of vitamins and micro minerals per kg of complete diet: vitamin A as retinyl acetate, 11,150 IU; vitamin D_3_ as cholecalciferol, 2,210 IU; vitamin E as DL-alpha tocopheryl acetate, 66 IU; vitamin K as menadione dimethylprimidinol bisulfite, 1.42 mg; thiamin as thiamine mononitrate, 1.10 mg; riboflavin, 6.59 mg; pyridoxine as pyridoxine hydrochloride, 1.00 mg; vitamin B_12_, 0.03 mg; D-pantothenic acid as D-calcium pantothenate, 23.6 mg; niacin, 44.1 mg; folic acid, 1.59 mg; biotin, 0.44 mg; Cu, 20 mg as copper sulfate; Fe, 125 mg as iron sulfate; I, 1.26 mg as ethylenediamine dihydriodide; Mn, 60.2 mg as manganous sulfate; Se, 0.30 mg as sodium selenite and selenium yeast; and Zn, 125.1 mg as zinc sulfate

^c^The phytase concentrate contained 5,000 units of phytase/g (Quantum Blue, AB Vista, Marlborough, UK). Five separate premixes were prepared by mixing 80, 160, 320, 640, or 1,280 g of the phytase concentrate with 7.920, 7.840, 7.680, 7.360, or 6.720 kg of ground corn. Each of these premixes were added to their respective diets by 0.50% to create final diets containing 250, 500, 1,000, 2,000, or 4,000 FTU/kg

^d^*AEE* Acid hydrolyzed ether extract

^e^*IP* Inositol phosphate. The concentrations of IP3 and inositol in the experimental diets were undetectable

### Sample collection and bone measurements

The day before the start of feeding experimental diets (d −1), pigs were weighed and a blood sample was collected by jugular venipuncture in lithium-heparin-containing tubes. Blood samples were centrifuged at 1,500 × *g* at 4 ºC immediately after collection and 2 samples of plasma were harvested from each pig and stored at −20 ºC. Pigs were fed experimental diets for 23 d. The initial 18 d was an adaptation period to the diets; an 18-day adaptation period was used in this experiment because of the hypothesis that pigs need more than 5 d to adapt to phytase to be able to increase AA digestibility. In the morning of d 19 and 20, fecal samples were collected via one-time anal stimulation and quantities between 50 and 100 g of fresh feces were collected each day. On d 21 and 22, ileal digesta were collected for 9 h/d following standard procedures [17]. Fecal and ileal digesta samples were stored at −20 °C immediately after collection.

On d 23, pigs were euthanized via captive bolt stunning and a blood sample and the right rear foot were collected. All collected feet were autoclaved at 125 ºC for 55 min and the 3^rd^ and 4^th^ metatarsals were identified, removed, and cleaned of soft tissue. Metatarsals were dried overnight at 105 ºC and soaked for 72 h in petroleum ether under a chemical hood to remove marrow and fat. Bones were then dried at 135 ºC for 2 h and ashed at 600 ºC for 16 h following the procedure described by Lee et al. [21].

### Sample analysis

Ileal digesta samples were thawed, mixed within animal and diet, and a sub-sample was collected. Ileal digesta and fecal samples were lyophilized [22] and ingredient, diet, ileal digesta, and fecal samples were finely ground. Ingredients and diets were analyzed for dry matter by oven drying at 135 °C for 2 h (Method 930.15; [23]) and for ash at 600 °C at 2 h (Method 942.05; [23]). Ingredient, diet, ileal digesta, and fecal samples were analyzed for Ca and P by inductively coupled plasma-optical emission spectrometry (Method 985.01 A, B, and C; [23]) after dry ash preparation (Method 942.05; [23]) followed by wet digestion with nitric acid (Method 3050 B; [24]). One of the plasma samples collected on d −1 and on d 23 was also analyzed for Ca and P by inductively coupled plasma-optical emission spectrometry, but after wet ash sample preparation (Method 975.03 B (b); [23]). Diet and fecal samples were analyzed for K, Mg, and Na, and ileal digesta samples were also analyzed for K. Diet and ileal digesta samples were analyzed for AA (Method 982.30 E [a, b, c]; [23]) using a Hitachi AA Analyzer (Model No. L8800; Hitachi High Technologies America, Inc., Pleasanton, CA, USA). Corn, SBM, diet, and ileal digesta samples were analyzed for N by the combustion procedure (method 990.03; [23]) using a LECO FP628 (LECO Corp., Saint Joseph, MI, USA) and crude protein (CP) was calculated as N × 6.25. Corn, SBM, diet, and fecal samples were analyzed for gross energy (GE) using an isoperibol bomb calorimeter (Model 6400, Parr Instruments, Moline, IL, USA). Corn, SBM, and diets were analyzed for acid hydrolyzed ether extract (AEE; Method 2003.06; [23]) using an Ankom^HCl^ Hydrolysis System followed by an Ankom^XT15^ Extractor (Ankom Technology, Macedon, NY, USA) and for phytate-bound P by wet chemistry using the Megazyme kit (Megazyme Inc., Chicago, IL, USA). Diet, fecal, and ileal digesta samples were also analyzed for Cr (Method 990.08; [23]) and these samples were analyzed at the University of East Anglia, School of Biological Sciences (UK) for inositol and IP esters using high-performance ion chromatography-based techniques as described by Walk et al. [25]. Diets were analyzed for phytase activity by the enzyme-linked immunosorbent assay method using Quantiplate Kits for Quantum (AB Vista, Plantation, FL, USA). From the second sample of plasma collected on d −1 and on d 23, 0.5 mL were transferred to a 2-mL centrifuge tube that contained 1 mL perchloric acid (1 mol/L). Samples were then stored at 4 °C for 30 min, centrifuged using a refrigerated centrifuge (Eppendorf Centrifuge 5427 R; Eppendorf AG, Hamburg, Germany) at 4 °C and 17,500 × *g* for 10 min. The supernatant was extracted using a 5-mL capacity sterile syringe (Fisherbrand™; Fisher Scientific, Waltham, MA, USA) with a needle attached, and the needle was then replaced with a syringe filter (Kinesis Polytetrafluoroethylene Syringe Filters; Cole-Parmer, Vernon Hills, IL, USA). The content was discharged into another tube, and this sample was analyzed for inositol as for diet, fecal, and ileal digesta samples.

### Calculations and statistical analyses

The percentage of phytate in corn and SBM was calculated by dividing the analyzed phytate-bound P by 0.282 [20], and non-phytate P was calculated by subtracting the amount of phytate-bound P from total P. The apparent ileal digestibility (AID) of CP, AA, Ca, P, and K and the apparent total tract digestibility (ATTD) of GE, Ca, P, K, Mg, and Na in experimental diets were calculated as previously outlined [26]:

$$\text{Digestibility of nutrients, }\% = \left[\text{1}-\left(\frac{\text{nutrient in sample}}{\text{nutrient in diet}}\right)\;\times\;\left(\frac{\text{marker in diet}}{\text{marker in sample}}\right)\right]\,\;\times\;100$$

where digestibility is the AID or ATTD of nutrients or energy, nutrient in sample is the nutrient or energy concentration in ileal digesta or fecal samples and nutrient in diet is the concentration of nutrient or energy in the diet. Marker in diet and marker in sample are the concentration of Cr in diets and ileal digesta or fecal samples, respectively. Bone ash percentage was calculated by dividing the quantity (g) of bone ash by the quantity of fat-free dried bone and multiplying by 100.

Normality of residuals and homogeneity of variances were tested using the INFLUENCE, GPLOT, and UNIVARIATE procedures of SAS (SAS® 9.4 SQL Procedure User’s Guide, 4th ed. SAS Inst. Inc., Cary, NC, USA). Data for phytate degradation, concentration and percentage of bone ash, plasma Ca, P, and inositol, and digestibility of energy and nutrients were analyzed using the PROC MIXED procedure of SAS with the experimental unit being the pig. The model included the main effect of diet phytase level and the random effect of replicate. For all parameters, contrast statements were used to determine linear and quadratic effects of phytase inclusion level. Coefficients for the unevenly spaced linear contrasts were obtained using the PROC IML procedure of SAS. Data for blood metabolites obtained on d −1 was used as covariate to analyze blood data from d 23. If a linear or quadratic effect of phytase on phytate degradation, bone ash, or Ca and P digestibility was observed, a broken line analysis was conducted using the analyzed values for phytase and the NLIN procedure of SAS. The single-slope model was *y* = *L* + *U* × *(R − x)*, where (R* − x*) is zero when *x* > R [27]. The breakpoint value is R, the asymptote for the first segment is L, and the slope for the line segment is U [27]. A *t*-test was used to test the null hypothesis that the difference between AID and ATTD values for Ca, P, and K was equal to zero, and the PROC CORR procedure of SAS was used to determine the correlation between ATTD of P and bone ash. Treatment means were calculated using the LSMEANS statement in SAS. Statistical significance and tendency were considered at *P* < 0.05 and 0.05 ≤ *P* < 0.10, respectively.

## Results

One pig fed the diet with no phytase died 5 d after the start of feeding experimental diets. This pig was replaced by an extra pig that was cannulated at the same time as the pig that died. The remaining pigs consumed their diets without apparent problems and no health problems were observed during the experiment. The average final body weight of pigs was 29.44 ± 3.44 kg.

No effect of inclusion level of dietary phytase on the AID of Arg, Ile, Leu, Phe, Val, Ala, Cys, and Glu in diets was observed, but increasing concentration of phytase resulted in increased (linear and quadratic; *P* < 0.05) AID of CP and Trp (Table 3). The AID of Lys, Thr, and Asp linearly increased (*P* < 0.05) whereas the AID of Ser quadratically increased (*P* < 0.05) as phytase inclusion level increased in diets. There was also a tendency (*P* < 0.10) for an increase in the AID of Met, Gly, and Tyr (linear) and in the AID of His, Thr, Asp, and Gly (quadratic) as the dietary level of phytase increased.

**Table 3:** Apparent ileal digestibility of crude protein (CP) and amino acids (AA) in diets containing 0, 250, 500, 1,000, 2,000, or 4,000 units of microbial phytase (FTU)/kg of complete diet^a^

Item, %	Phytase, FTU/kg						SEM	*P-*value	
	**0**	**250**	**500**	**1,000**	**2,000**	**4,000**		**Linear**	**Quadratic**
CP	70.6	73.2	75.7	75.6	77.9	76.4	1.66	0.030	0.026
Indispensable AA									
Arg	89.5	89.5	90.2	90.1	90.3	90.4	0.61	0.287	0.505
His	79.4	80.3	82.7	82.4	82.6	81.2	1.32	0.544	0.087
Ile	80.5	80.4	82.2	82.6	83.1	82.5	1.18	0.190	0.161
Leu	80.3	80.1	82.3	82.6	82.5	81.7	1.30	0.463	0.174
Lys	82.7	84.7	86.2	85.8	85.7	88.1	1.10	0.008	0.593
Met	88.9	87.5	88.6	89.2	89.2	89.8	0.78	0.076	0.803
Phe	81.8	80.7	82.2	82.6	83.0	83.2	0.96	0.119	0.455
Thr	70.3	71.1	73.1	74.8	76.2	75.1	1.73	0.033	0.058
Trp	78.1	81.0	83.2	84.0	86.1	83.8	1.37	0.011	0.002
Val	76.4	76.7	79.0	79.3	80.1	78.8	1.50	0.241	0.103
Mean	80.8	81.3	83.2	83.4	83.8	83.1	1.19	0.196	0.116
Dispensable AA									
Ala	74.3	74.1	77.7	77.4	77.8	77.4	1.53	0.151	0.153
Asp	74.6	74.8	78.3	78.1	79.1	78.7	1.43	0.041	0.076
Cys	53.4	54.2	60.2	57.1	60.8	59.1	3.19	0.211	0.234
Glu	78.0	78.3	82.5	79.6	81.0	80.6	1.76	0.412	0.406
Gly	58.9	62.5	68.5	66.5	67.3	67.3	2.53	0.079	0.093
Ser	75.6	76.6	79.2	80.0	81.0	79.7	1.53	0.061	0.035
Tyr	81.3	80.4	82.2	82.6	83.1	83.6	1.07	0.050	0.405
Mean	74.1	74.9	78.7	77.4	78.5	78.1	1.61	0.124	0.141
All AA	77.4	78.1	81.0	80.4	81.1	80.6	1.36	0.143	0.121

^a^Data are least square means of 5 or 6 observations

**Fig. 1:**
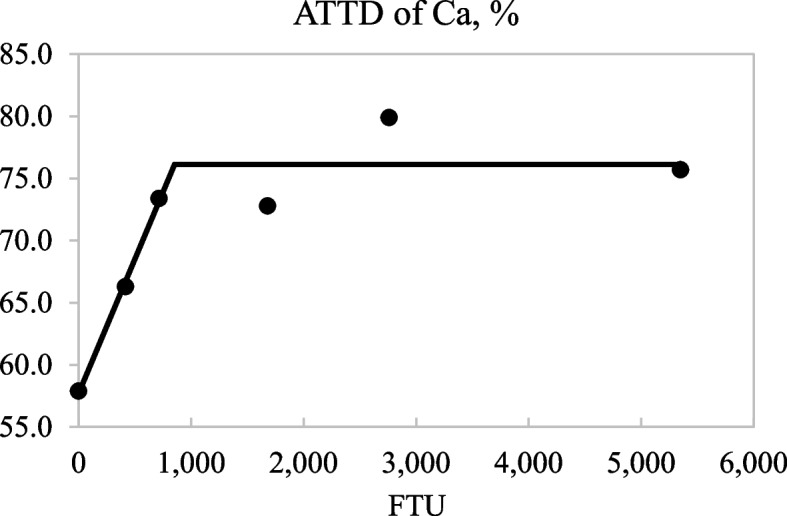
Fitted linear broken-line plots of the average apparent total tract digestibility (ATTD) of Ca in diets as a function of phytase inclusion level. The optimum concentration of phytase determined by linear broken-line model was 853 (SE = 168.7) units of microbial phytase (FTU)/kg of feed; [*Y* = 76.1 − 0.022 × (853 − *X*)] where *X* is less than 853, with *r*^2^ = 0.915 and *P* < 0.05. The SE for the estimates of the intercept and second parameter were 1.69 and 0.006, respectively

**Fig. 2:**
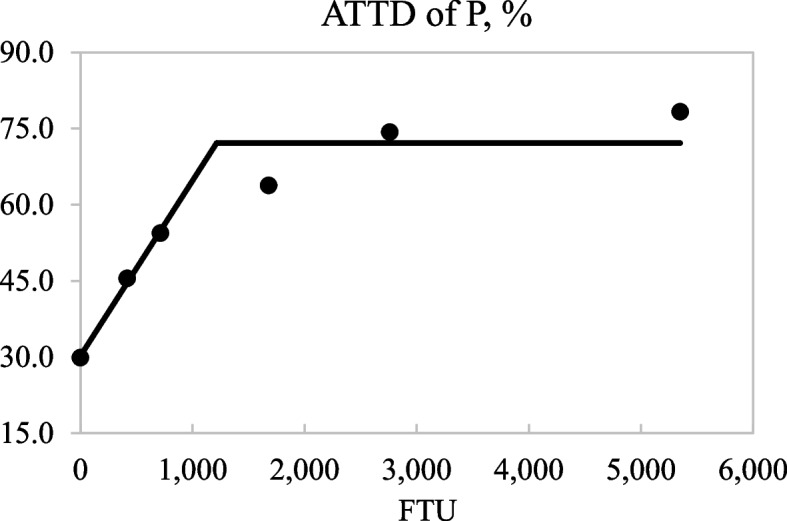
Fitted linear broken-line plots of the average apparent total tract digestibility (ATTD) of P in diets as a function of phytase inclusion level. The optimum concentration of phytase determined by linear broken-line model was 1,215 (SE = 328.1) units of microbial phytase (FTU)/kg of feed; [*Y* = 72.1 − 0.035 × (1,215 − *X*)] where *X* is less than 1,215, with *r*^2^ = 0.932 and *P* < 0.05. The SE for the estimates of the intercept and second parameter were 3.55 and 0.012, respectively

There was a linear and quadratic increase (*P* < 0.05) in the AID of Ca and P and the ATTD of Ca, P, and K as the inclusion level of phytase increased in diets (Table 4). The broken-line analyses indicated that 853 and 1,215 FTU/kg were needed to maximize the ATTD of Ca (*P* < 0.05; Fig. 1) and P (*P* < 0.05; Fig. 2), respectively. The AID of K linearly increased (*P* < 0.05) and tended (*P* < 0.10) to quadratically increase with increasing dietary concentrations of phytase. Likewise, there was a linear increase (*P* < 0.05) in the ATTD of Na in diets as phytase inclusion increased, but no effect of the level of phytase in diets was observed for the ATTD of GE or Mg. Differences between AID and ATTD of Ca differed (*P* < 0.05) from zero, but for P or K, no differences between AID and ATTD values were observed (Table 5).

**Table 4:** Digestibility of gross energy (GE) and minerals in diets containing 0, 250, 500, 1,000, 2,000, or 4,000 units of microbial phytase (FTU)/kg of complete diet^a^

Item, %	Phytase, FTU/kg						SEM	*P-*value	
	**0**	**250**	**500**	**1,000**	**2,000**	**4,000**		**Linear**	**Quadratic**
Apparent ileal digestibility									
Ca	61.6	74.2	76.7	77.1	80.7	76.8	1.99	0.001	< 0.001
P	27.1	45.1	56.7	63.0	75.5	80.0	1.43	< 0.001	< 0.001
K	81.1	83.6	85.1	83.8	87.0	86.7	1.18	0.003	0.072
Apparent total tract digestibility									
GE	82.7	83.2	84.3	82.2	83.9	81.8	1.27	0.415	0.439
Ca	57.9	66.3	73.4	72.8	79.9	75.7	3.09	< 0.001	< 0.001
P	29.9	45.5	54.4	63.8	74.3	78.3	3.07	< 0.001	< 0.001
K	79.4	83.3	85.2	87.0	89.5	88.2	1.46	< 0.001	< 0.001
Mg	21.4	18.1	21.5	24.7	27.9	26.6	4.56	0.135	0.333
Na	83.3	82.5	83.5	84.3	85.7	89.7	2.54	0.035	0.840

^a^Data are least square means of 5 or 6 observations

**Table 5:** Comparison between values for ileal and total tract digestibility of Ca, P, and K in diets^a^

**Item**	**Digestibility, %**			**SEM**	***P-*value**
	**Ileal**	**Total tract**	**Difference**		
Ca	74.4	70.4	4.11	1.28	0.003
P	57.8	57.1	0.44	1.04	0.675
K	84.6	85.4	- 0.79	0.71	0.275

^a^Data are least square means of 33, 32, and 35 observations for Ca, P, and K, respectively

**Fig. 3:**
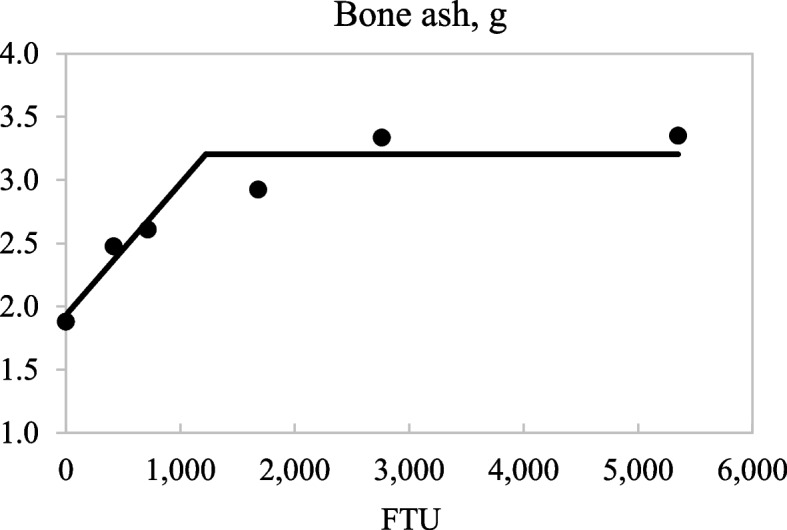
Fitted linear broken-line plots of the average concentration of bone ash (g) as a function of phytase inclusion level. The optimum concentration of phytase determined by linear broken-line model was 1,222 (SE = 378.1) units of microbial phytase (FTU)/kg of feed; [*Y* = 3.20 − 0.001 × (1,222 − *X*)] where *X* is less than 1,222, with *r*^2^ = 0.914 and *P* < 0.05. The SE for the estimates of the intercept and second parameter were 0.123 and 0.0004, respectively

There was no effect of dietary phytase on the concentration of Ca in plasma of pigs on d 23, but increased concentrations of plasma P (linear and quadratic; *P* < 0.05) and plasma inositol (linear; *P* < 0.05) were observed as dietary phytase increased (Table 6). The concentration and percentage of bone ash linearly and quadratically increased (*P* < 0.05) with increasing dietary phytase. The broken-line analyses indicated that 1,222 FTU/kg were needed to maximize the concentration of bone ash (*P* < 0.05; Fig. 3).

**Table 6:** Concentration of Ca, P, and inositol in plasma and quantity and concentration of bone ash in pigs fed diets containing 0, 250, 500, 1,000, 2,000, or 4,000 units of microbial phytase (FTU)/kg of complete diet^a^

Item	Phytase, FTU/kg						SEM	*P-*value	
	**0**	**250**	**500**	**1,000**	**2,000**	**4,000**		**Linear**	**Quadratic**
d −1									
Plasma									
Ca, mg/dL	9.87	9.78	9.88	9.53	9.80	10.00	-	-	-
P, mg/dL	10.32	10.23	9.53	9.82	10.48	10.53	-	-	-
Inositol, μmol/L	8.5	6.8	7.3	11.5	10.5	7.2	-	-	-
d 23									
Plasma^b^									
Ca, mg/dL	11.10	10.84	11.35	10.94	11.28	11.75	0.49	0.233	0.762
P, mg/dL	9.89	12.16	13.64	14.27	15.49	16.28	0.51	< 0.001	< 0.001
Inositol, μmol/L	80.7	81.1	97.4	86.4	103.0	112.1	10.9	0.024	0.716
Bone									
Ash, g	1.88	2.48	2.61	2.92	3.34	3.35	0.09	< 0.001	< 0.001
Ash, %	48.3	50.4	53.1	53.9	55.2	55.5	0.60	< 0.001	< 0.001

^a^Data are least square means of 6 observations

^b^Data from d −1 were used as a covariate for data obtained on d 23

**Fig. 4:**
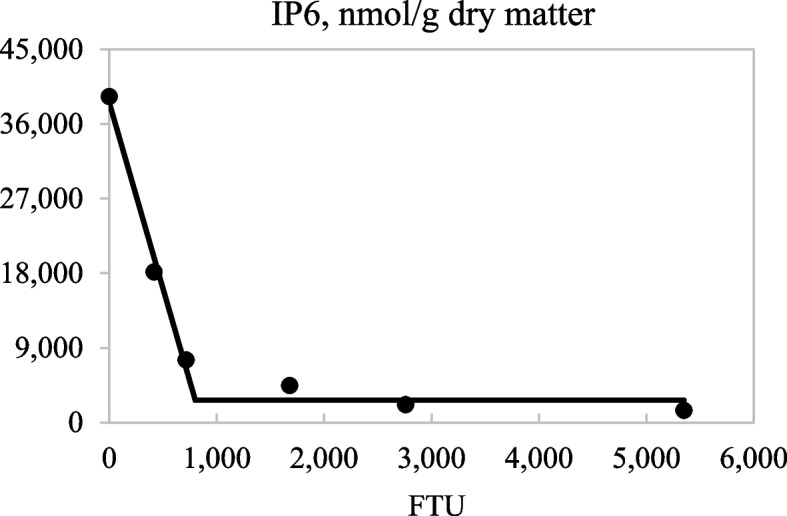
Fitted linear broken-line plots of the average concentration of ileal IP6 (nmol/g dry matter) as a function of phytase inclusion level. The optimum concentration of phytase determined by linear broken-line model was 801 (SE = 46.3) units of microbial phytase (FTU)/kg of feed; [*Y* = 2,690 + 44.8 × (801 − *X*)] where X is less than 801, with *r*^2^ = 0.991 and *P* < 0.01. The SE for the estimates of the intercept and second parameter were 1,027 and 3.50, respectively

**Fig. 5:**
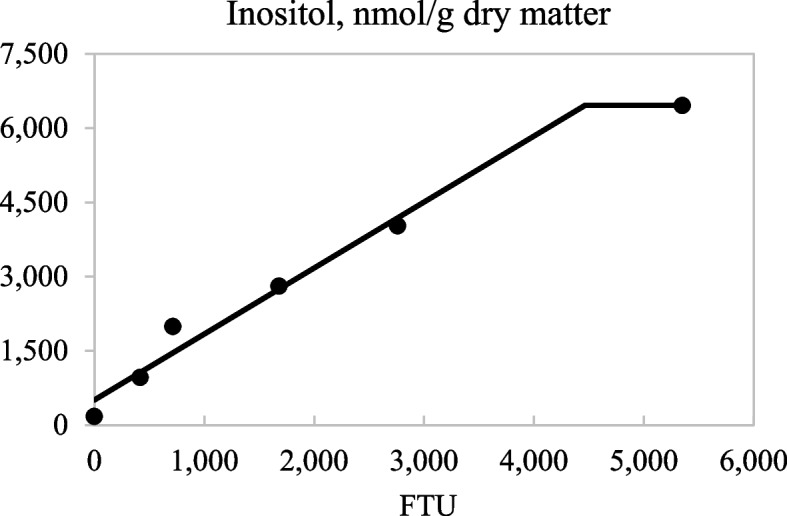
Fitted linear broken-line plots of the average concentration of ileal inositol (nmol/g dry matter) as a function of phytase inclusion level. The optimum concentration of phytase determined by linear broken-line model was 4,464 (SE = 531.0) units of microbial phytase (FTU)/kg of feed; [*Y* = 6,458 − 1.33 × (4,464 − *X*)] where *X* is less than 4,464, with *r*^2^ = 0.983 and *P* < 0.01. The SE for the estimates of the intercept and second parameter were 379.5 and 0.171, respectively

**Fig. 6:**
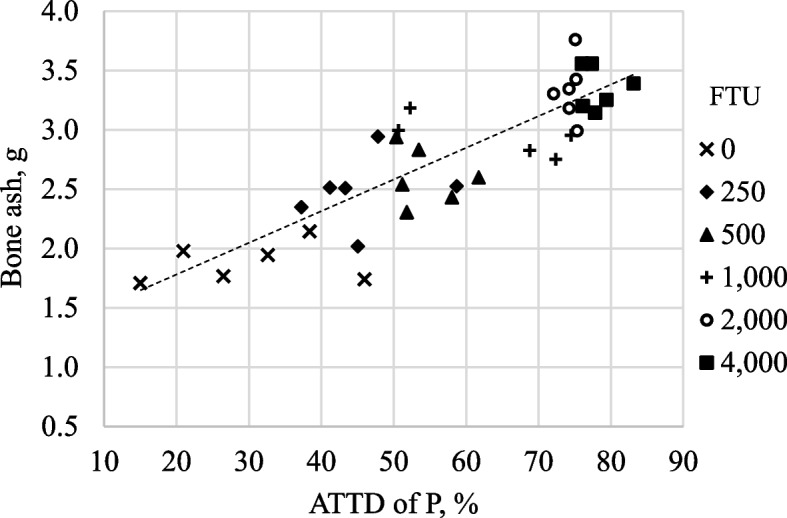
Correlation between ATTD of P and bone ash (g/bone). Model: *Y* = 0.0263*X* + 1.2465; *r*^2^ = 0.729 and *P* < 0.001

Concentrations of IP6 and IP5 in ileal digesta decreased (linear and quadratic; *P* < 0.05) as the inclusion of dietary phytase increased from 0 to 4,000 FTU/kg (Table 7). Increasing dietary phytase also resulted in reduced (linear; *P* < 0.05) concentrations of ileal IP4 and IP3, but increased (linear; *P* < 0.05) concentrations of ileal inositol. The broken-line analyses indicated that 801 and 4,464 FTU/kg were needed to maximize IP6 degradation (*P* < 0.05; Fig. 4) and inositol release in ileal digesta (*P* < 0.05; Fig. 5), respectively. There was a linear decrease (*P* < 0.05) in the concentration of fecal IP5 and a tendency for a quadratic increase (*P* < 0.10) in fecal IP4 with increasing inclusion of phytase, but no effect of dietary phytase on the concentration of IP6 in feces was observed. There was a strong linear correlation (*r*^2^ = 0.729; *P* < 0.01) between ATTD of P and the concentration of bone ash (Fig. 6).

**Table 7:** Inositol phosphate (IP) esters and inositol (nmol/g dry matter) in ileal digesta and fecal samples from pigs fed diets containing 0, 250, 500, 1,000, 2,000, and 4,000 units of microbial phytase (FTU)/kg of complete diet^a^

Item	Phytase, FTU/kg						SEM	*P-*value	
	**0**	**250**	**500**	**1,000**	**2,000**	**4,000**		**Linear**	**Quadratic**
Ileal digesta samples									
IP6	39,298	18,147	7,546	4,460	2,136	1,474	1,597	< 0.001	< 0.001
IP5	5,223	3,822	1,703	1,006	334	225	522	< 0.001	< 0.001
IP4	1,253	7,312	5,749	5,700	2,580	822	1,182	0.003	0.111
IP3	727	3,569	2,541	2,618	1,109	543	583	0.005	0.292
Inositol	173	961	1,990	2,808	4,026	6,458	570	< 0.001	0.135
Fecal samples^b^									
IP6	775	509	606	968	670	741	136	0.683	0.641
IP5	149	73	59	97	58	48	24	0.041	0.295
IP4	24	33	58	62	31	26	10	0.258	0.076

^a^Data are least square means of 6 observations

^b^The concentrations of IP3 and inositol in all samples were undetectable

## Discussion

The analyzed concentrations of GE and nutrients were consistent among diets and in agreement with formulated values (Table 2). However, analyzed phytase concentrations were 35% to 65% greater than formulated values, which is likely a result of an overage of phytase in the phytase concentrate used. Phytase companies often include an overage in the concentrate to account for a potential loss of activity during storage, diet preparation, or feed processing (e.g., pelleting). In this experiment, diets were fed as mash, which may explain the high recoveries of phytase. The small differences among diets in analyzed phytate-P is likely the result of analytical inaccuracies because all diets were similar and contained the same quantities of phytate and P.

Phytate and lower phytate esters have anti-nutritional effects in pigs because of binding of dietary nutrients including minerals and AA, which results in reduced nutrient digestibility [5]. Super-dosing of phytase is a strategy to reduce the anti-nutritional effects of phytate in diets by degradation of not only phytate, but also lower phytate esters in the small intestine of pigs [15]. The subsequent increase in inositol release is also considered a beneficial effect of super-dosing of phytase because of the role of inositol in metabolic processes [28]. However, although positive effects of phytase on phytate degradation, mineral digestibility, and growth performance of pigs is usually observed [13, 29], effects of high levels of phytase on energy and AA digestibility are not consistent [10, 14, 30].

The observed increase in the AID of CP, Lys, Trp, and Thr, as well as the tendency for an increased AID of Met, as dietary phytase increased from 0 to 4,000 FTU/kg indicates that super-dosing of phytase increases the digestibility of the 4 first limiting AA in corn-SBM diets fed to pigs. The linear increase in the AID of Lys and Met indicates that more than 4,000 FTU/kg may be needed to maximize AID of Lys and Met, whereas the observation that the increase in the AID of Thr and Trp was quadratic indicates that 2,000 FTU/kg was sufficient to maximize AID of these two AA. However, besides a tendency for a quadratic increase in the AID of His as phytase increased in diets, no other indispensable AA, nor the average of all indispensable AA, was influenced by phytase. The observation that increasing levels of phytase increased or tended to increase the AID of Asp, Ser, and Tyr is in agreement with results from experiments using dietary phytase from 0 to 2,000 FTU/kg [14, 30]. However, the AID of CP and AA was not affected by dietary phytase of up to 3,000 FTU/kg in other experiments [10, 15, 16, 31]. It is not clear why results of the present experiment were different from some published data, but it is possible that the lighter body weight of pigs used in the current experiment compared with pigs used in some other experiments [10, 15] or the greater levels of phytase used in this experiment contributed to this difference. However, the longer adaptation period used in this experiment compared with previous experiments may also have influenced the results, but more research is needed to verify this hypothesis. The numerical, but non-significant, increases in AID of 3.0, 4.4, and 3.7 percentage units for the average of indispensable, dispensable, and all AA that were calculated in this experiment as a result of using 2,000 FTU/kg in diets are in agreement with data from Cowieson et al. [32] who reported an average increase of 2.8 percentage units in AID of all AA from a review of 28 publications. The fact that all AA in the diet containing 2,000 FTU/kg had AID values that were numerical greater than in the diet without phytase indicates that the differences were not random. However, it is likely that more replications than typically used in digestibility experiments are needed to verify effects of phytase on AID of AA. Indeed, significant increases in the AID of 9 of the 10 indispensable AA was demonstrated in an experiment where 34 to 36 replicates per treatment were used, which supports the hypothesis that a high number of replicates are needed to demonstrate positive effects of phytase on AA digestibility [33].

The observation that increasing levels of phytase did not increase the ATTD of GE is in agreement with published data [10, 14–16], but in contrast with data from Arredondo et al. [13] who reported a positive effect of phytase on the ATTD of GE. The reason for the inconsistency in the effects of phytase on ATTD of GE may be related to difference in the phytases used.

The quadratic increase in the ATTD of P and Ca as phytase supplementation increased in diets is well documented [10, 11, 13, 34]. The broken-line regression analysis indicated that inclusion of phytase above 853 and 1,215 FTU/kg did not further improve the ATTD of Ca and P, respectively, which is in agreement with Almeida et al. [11] and Arredondo et al. [13]. The observed increase in the ATTD of K and Na concurs with data from She et al. [10] and Arredondo et al. [13], but the lack of a phytase effect on the ATTD of Mg is in contrast with published data, and was not expected because phytate is present in plants as Ca, Mg, and K mixtures [35]. Overall, the hypothesis that pigs allowed an 18-day adaptation period to a phytase containing diet would have greater digestibility of minerals, energy, and AA if microbial phytase was included in the diet by up to 4,000 FTU was partly accepted due to the increased AID of some AA and of all analyzed minerals except Mg. However, the hypothesis that microbial phytase can also increase the ATTD of GE was rejected. In addition, because phytase effects on mineral digestibility are ingredient and diet specific [36, 37] results may be different in diets with a different ingredient composition.

No differences have been observed between values for AID and ATTD of Ca and P in pigs, which indicates that there is no net absorption of these minerals in the hindgut [38, 39]. Data from this experiment concurs with previous results as indicated by the lack of differences between AID and ATTD values for P and K, whereas a small, but significant, reduction in ATTD of Ca compared with AID of Ca was observed. It is not clear why results for Ca in this experiment were different from previous experiments, but the lower value for ATTD compared with AID of Ca indicates that small amounts of Ca may have been secreted into the hindgut of the pigs. Although it was concluded by Bohlke et al. [38] that there was no difference between AID and ATTD of Ca, values for ATTD of Ca were actually slightly greater than AID values in that experiment as well, but the differences were not significant. There is, therefore, a need for conducting more research to address this question as it appears that it is possible there is a small secretion of endogenous Ca into the hindgut.

The observation that phytase level did not affect the concentration of Ca in plasma is in agreement with data from Cowieson et al. [40] and Mesina et al. [15]. The constant concentration of plasma Ca between 10.9 and 11.8 mg/dL regardless of ATTD of Ca is a result of the regulation of Ca homeostasis by systemic hormones [41]. The observed increase in the concentration of P and inositol in plasma as dietary phytase increased concurs with previous data from pigs [15, 40] and demonstrate that plasma P concentration is not as strictly regulated as is the case for plasma Ca concentration. In fact, it is evident that as more phytase was included in the diet, more phosphate was removed from phytate and phytate esters, which resulted in more P being absorbed, increasing plasma P concentrations, but at the same time, increasing the amount of phytate being fully degraded. The observed increase in plasma inositol from 81 μmol/L when no phytase was used to 97 μmol/L upon supplementation of 500 FTU/kg of phytase indicates that even at a low inclusion of phytase, full degradation of some phytate molecules in the gastrointestinal tract of pigs takes place and as more phytase was included in the diet, more inositol was absorbed, increasing plasma inositol and indicating further degradation of phytate molecules in the intestinal tract. This degradation of phytate is also the reason for the observed increase in ATTD of P and most of the divalent cations, because if there is no phytate, P cannot be bound to the inositol ring and if P is not bound to inositol, the cations cannot be bound either. Inositol is involved in several metabolic processes as part of phosphatidylinositol phosphates, which are mediators in cellular signaling to activate protein B kinase and stimulate glucose uptake and protein synthesis [42]. Therefore, the increased release and absorption of inositol may be beneficial for pigs as indicated by the improved growth performance of newly weaned pigs upon inositol or phytase supplementation [43], and the increased abundance of glucose transporter type 4 in muscle of growing pigs fed diets with 2,000 FTU/kg [29].

The quadratic increase in bone ash as phytase increased in diets is in agreement with previous data from pigs [44, 45], and is the result of the increased ATTD of Ca and P as phytase was added to the diets. The breakpoint in the linear-plateau analysis indicates that there is no improvement in the concentration of bone ash after addition of 1,222 FTU/kg. This result concurs with the breakpoint at 1,215 FTU/kg observed for the ATTD of P and indicates that P was the limiting nutrient for bone synthesis in these pigs. As a consequence, the hypothesis that increased concentrations of microbial phytase in diets for pigs would increase plasma concentrations of P and inositol and also increase bone ash was confirmed.

The reduced concentrations of IP6 and IP5 in ileal digesta along with the increase and then decrease in the concentrations of IP4 and IP3 as phytase inclusion increased from 0 to 4,000 FTU/kg is a result of the stepwise hydrolysis of phytate by phytase to release phosphate from inositol. These results are in agreement with data from Laird et al. [46], Mesina et al. [15], and Rosenfelder-Kuon et al. [16] using different diet ingredient combinations. The concentrations of IP esters and inositol observed in this experiment are lower than those reported by Mesina et al. [15], which is a result of a greater substrate supply in diets with canola meal because there is a greater concentration of ileal IP6, IP4, and inositol in pigs if canola meal is included in diets [16]. The observation that after the inclusion of 800 FTU/kg, there was no improvement in IP6 degradation, but the breakpoint where inositol release is no longer improved was 4,464 FTU/kg, indicates that IP6 concentration does not represent the complete destruction of phytate because of the progressive degradation of IP6 and IP5 into lower esters and inositol as dietary phytase increased. However, the observation that the breakpoint for P digestibility and bone ash is at around 1,200 FTU/kg indicates that the P released by phytase after this point is not utilized for bone ash synthesis, which may be a result of formation of Ca-P complexes in the small intestine of pigs that makes P unavailable [47]. It is also possible that some of the P is released too far down the intestinal tract to be fully absorbed, but additional research is needed to test this hypothesis. The fact that there was a strong positive correlation between ATTD of P and bone ash confirms that P was the limiting factor for bone ash synthesis. Although ATTD of Ca also increased as dietary phytase increased, the correlation with bone ash was much weaker (*r*^2^ = 0.281; data not shown), which is likely because the impact of phytase on P digestibility is much greater than on Ca digestibility. However, bone mineralization can only take place if both Ca and P are present in bone cells at the same time [41].

The reduced concentrations of IP esters in feces compared with ileal digesta and the limited effect of dietary phytase on fecal IP esters is in agreement with Mesina et al. [15] and indicates that phytate that is not degraded in the small intestine by exogenous phytase is degraded in the hindgut by phytase synthesized by intestinal microbes [48]. It is likely that the majority of the phytate is degraded in the cecum, but to our knowledge, degradation of phytate in the cecum has not been quantified and this speculation, therefore, needs to be experimentally verified. However, as observed in this and previous experiments, phytate degradation beyond the ileum is not relevant for pigs, because there is no net absorption of Ca and P in the hindgut of pigs [38, 39, 49]. As a consequence, phytate and IP esters should be determined at the distal ileum rather than in feces. Nevertheless, the hypothesis that the maximum degradation of phytate requires more phytase than what is needed to maximize digestibility of P was confirmed.

## Conclusions

Increasing microbial phytase from 0 to 4,000 FTU/kg in corn-SBM diets fed to young pigs using an 18-day adaptation period increased digestibility of some AA, Ca, P, K, and Na, but did not impact digestibility of Mg or energy. However, plasma P, plasma inositol, and the concentration of bone ash increased as dietary phytase increased, indicating that degradation of phytate and lower phytate esters results in increased Ca and P release from phytate, and increased absorption and retention of Ca and P. The observed increase in ileal and plasma inositol by phytase also indicates that phytase fully degraded at least some of the phytate, and thereby increased inositol release and absorption. Degradation of phytate in the hindgut of pigs by microbes was observed, but it is considered irrelevant because no net absorption of Ca and P in the large intestine takes place. At least 1,200 FTU/kg of phytase were needed to maximize P digestibility, but to fully degrade phytate, more than 4,000 FTU/kg of phytase were needed.

## Data Availability

All data generated or analyzed during this study are included in this published article.

## Abbreviations

AA: Amino acids
AEE: Acid hydrolyzed ether extract
AID: Apparent ileal digestibility
ATTD: Apparent total tract digestibility
CP: Crude protein
FTU: Phytase units
GE: Gross energy
IP: Inositol phosphate
SBM: Soybean meal
